# Increased mtDNA Abundance and Improved Function in Human Barth Syndrome Patient Fibroblasts Following AAV-*TAZ* Gene Delivery

**DOI:** 10.3390/ijms20143416

**Published:** 2019-07-11

**Authors:** Silveli Suzuki-Hatano, Mughil Sriramvenugopal, Manash Ramanathan, Meghan Soustek, Barry J. Byrne, W. Todd Cade, Peter B. Kang, Christina A. Pacak

**Affiliations:** 1 Department of Pediatrics, University of Florida College of Medicine, Gainesville, FL 32610, USA; 2 Department of Molecular Genetics and Microbiology, University of Florida College of Medicine, Gainesville, FL 32610, USA; 3 Program in Physical Therapy, Washington University School of Medicine, St. Louis, MO 63110, USA

**Keywords:** Barth syndrome, mitochondrial disease, mtDNA copy numbers, gene therapy, AAV, TMEM65, fibroblasts

## Abstract

Barth syndrome (BTHS) is a rare, X-linked, mitochondrial disorder caused by mutations in the gene encoding tafazzin. BTHS results in cardiomyopathy, muscle fatigue, and neutropenia in patients. Tafazzin is responsible for remodeling cardiolipin, a key structural lipid of the inner mitochondrial membrane. As symptoms can vary in severity amongst BTHS patients, we sought to compare mtDNA copy numbers, mitochondrial fragmentation, and functional parameters between primary dermal BTHS fibroblasts isolated from patients with two different mutations in the *TAZ* locus. To confirm cause‒effect relationships and further support the development of gene therapy for BTHS, we also characterized the BTHS cells following adeno-associated virus (AAV)-*TAZ* transduction. Our data show that, in response to AAV-*TAZ* transduction, these remarkably dynamic organelles show recovery of mtDNA copy numbers, mitochondrial structure, and mitochondrial function, providing additional evidence to support the therapeutic potential of AAV-mediated gene delivery for BTHS. This study also demonstrates the direct relationship between healthy mitochondrial membrane structure and maintenance of proper levels of mtDNA copy numbers.

## 1. Introduction

Barth syndrome (BTHS) is a rare mitochondrial disorder caused by recessive loss-of-function mutations in the nuclear-encoded gene *TAZ*, which encodes tafazzin. Tafazzin is an acyltransferase that is trafficked to the inner mitochondrial membrane (IMM) where it remodels monolysocardiolipin (MLCL) to mature cardiolipin (CL) [1,2]. A critical phospholipid, CL is involved in maintenance of mitochondrial membrane structure and fluidity, osmotic stability, and efficient electron transport chain (ETC) function [3,4,5,6]. In BTHS mitochondria, the increased MLCL/CL ratios result in an unstable IMM structure and ultimately, inefficient ETC-mediated ATP production [7,8,9]. Typical BTHS symptoms include cardioskeletal myopathy, impaired cardiac and skeletal muscle bioenergetics, neutropenia, and 3-methylglutaconic aciduria, with cardiomyopathy being the primary cause of death [10,11,12,13,14,15,16].

Two primary, non-SV40 transformed, dermal BTHS fibroblast lines that represent different mutations in the *TAZ* locus: BTHS1 (c.170G>T in exon 2—missense mutation), BTHS 2 (c.140-152del13 in exon 2—frameshift mutation), and 2 healthy control lines were used in this study [6,17,18]. Although the structural integrity of the IMM is known to be impaired in BTHS, investigations into levels of mtDNA copy numbers in BTHS and whether these levels successfully recover following a gene replacement strategy in BTHS patient-derived cells have not yet been performed.

Clinical BTHS standard-of-care currently involves administration of diuretics and ACE inhibitors and many patients eventually undergo cardiac transplantation. No disease-modifying therapies are available for BTHS patients. Thus, we have been working to develop and test an adeno-associated virus (AAV)-mediated gene therapy for delivery of the human *TAZ* gene. Recently, we demonstrated the ability of this approach to significantly improve heart and skeletal muscle function in a mouse model of BTHS using a wide variety of functional and quantitative proteomic analyses [19,20]. Here we demonstrate the potential of this therapy in the background of two different mutations in the *TAZ* locus and its direct impact on mtDNA copy numbers.

## 2. Results

### 2.1. BTHS Fibroblast Mitochondria Display Structural Alterations

The two BTHS fibroblasts lines used in this study represent either a missense mutation (BTHS1) or a deletion with frameshift leading to premature stop (BTHS2) (Table 1).

**Figure 1:**
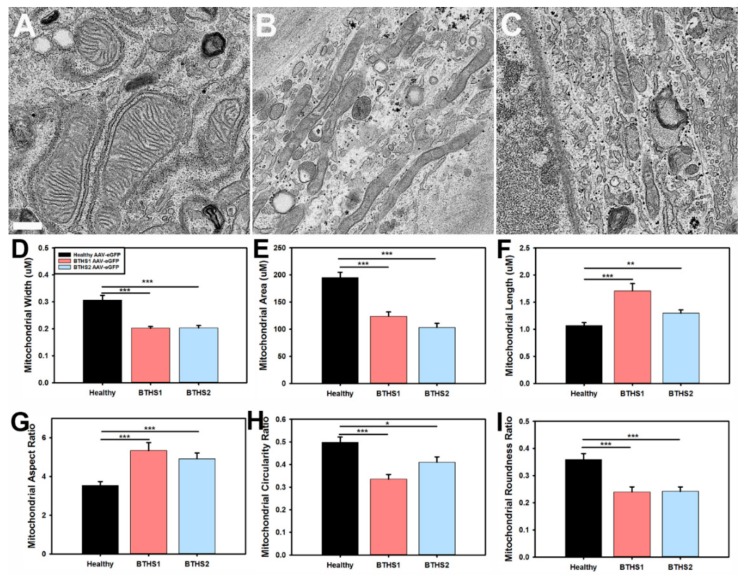
EM characterization of mitochondrial structures – Representative EM images from (A) healthy controls, (**B**) BTHS1, and (**C**) BTHS2 fibroblast lines used to perform mitochondrial contour evaluations (scale bar = 0.5 μm). Bar graphs representing mitochondrial (**D**) aspect ratios, (**E**) circularity ratios, (**F**) roundness ratios, (**G**) solidity ratios, (**H**) width, and (**I**) alterations in various aspects of mitochondrial contour in BTHS. (*n* ≥ 50 individual mitochondria for each measurement; data are presented as mean + std. err. * *p* ≤ 0.05, ** *p* ≤ 0.01, *** *p* ≤ 0.001.)

Electron microscopy (EM) was performed and revealed more-diffuse and less-organized cristae structures in both BTHS lines as compared to healthy controls (Figure 1A–C). Mitochondrial width and area were significantly decreased in both BTHS lines (Figure 1D,E), while length was significantly increased in both BTHS lines as compared to healthy controls (Figure 1F). Comparisons of mitochondrial contour ratio calculations between the different lines revealed a significant increase in aspect ratios, and a significant decrease in circularity and roundness ratios in the BTHS lines as compared to healthy controls (Figure 1G–I).

### 2.2. AAV-*TAZ* Administration Increases mtDNA Copy Numbers

**Figure 2:**
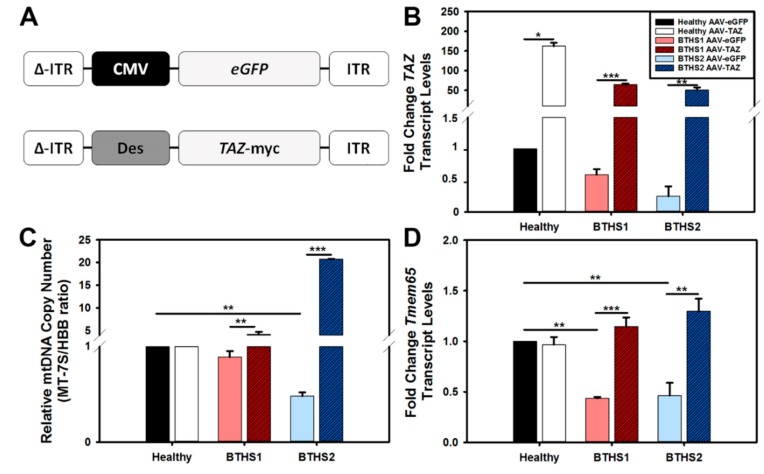
Vector design, *TAZ* gene expression, and mtDNA copy numbers – (**A**) Diagrams of the GFP marker gene control (top) and human *TAZ* containing AAV vectors used in this study. ΔITR on the left represents the double-stranded version of AAV inverse terminal repeats. (**B**) *TAZ* gene expression assay results demonstrate dramatic fold increase in expression over baseline for AAV-*TAZ*-treated cells. (**C**) Relative mtDNA copy numbers in BTHS lines following AAV-*eGFP* (control vector—light filled bars) or AAV-*TAZ* treatment (dark hashed bars) as compared to respective healthy control levels. (**D**) *TMEM65* gene expression results demonstrate significant increases in BTHS lines following AAV-*TAZ* gene delivery. *TAZ* and *TMEM65* gene expression levels were normalized to 18S expression for all samples. (Data are presented as mean + std. err. * *p* ≤ 0.05, ** *p* ≤ 0.01, *** *p* ≤ 0.001.)

Next, we sought to test how well we could transduce human dermal fibroblasts using the previously described triple tyrosine mutant (Y–F) AAV capsid known to efficiently transduce cells in vitro [21]. Both a control vector expressing eGFP and a therapeutic vector expressing *TAZ* were used to treat BTHS fibroblasts at a multiplicity of infection (m.o.i.) of 50,000 vector genomes (v.g.) per cell (Figure 2A). For this and all subsequent assays, cells were collected or assessed seven days post-AAV transduction. *TAZ* gene expression was found to have increased more than 50-fold in both BTHS lines following treatment with the AAV-*TAZ* vector as compared to cells treated with the same dose of the control vector (Figure 2B).

Evaluations of relative mtDNA copy numbers amongst healthy and BTHS lines revealed a significant decrease in mtDNA copy numbers in BTHS2 as compared to healthy controls (Figure 2C). There was a significant increase in mtDNA copy numbers in both BTHS lines following AAV-*TAZ* administration as compared to administration of the AAV-*eGFP* control vector. As we previously found significant downregulation of the TMEM65 protein and its transcription levels in hearts from a mouse model of BTHS, we also measured *TMEM65* gene expression in these BTHS fibroblast lines and found significantly decreased levels as compared to healthy controls (Figure 2D). In each case, *TMEM65* gene expression levels significantly increased following AAV-*TAZ* treatment.

### 2.3. Mitochondrial Fragmentation in BTHS

**Figure 3:**
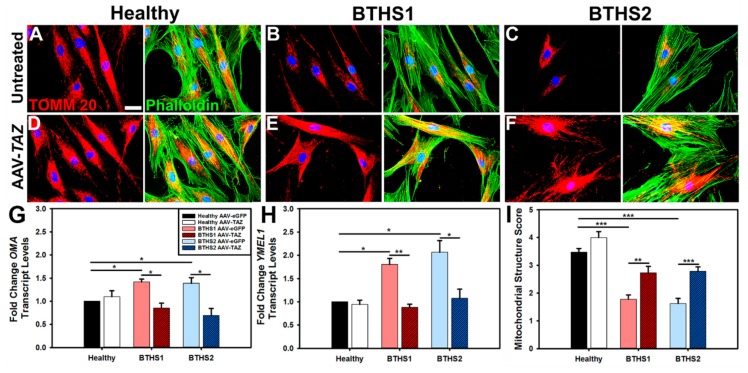
Mitochondrial fragmentation in BTHS is reduced following AAV-*TAZ* treatment – IF showing TOMM20 (red) staining for mitochondria, phalloidin (green) for cytoskeletal architecture, and DAPI (blue) for cell nuclei. Representative IF images from untreated (**A**) healthy control, (**B**) BTHS1, and (**C**) BTHS2 as well as AAV-*TAZ*-treated (**D**) healthy control, (**E**) BTHS1, and (**F**) BTHS2 fibroblast lines (scale bar = 5 μm). (**G**) *OMA*, or (**H**) *YME1L1* levels for each cell line following administration of either the control AAV vector (light filled bars) or AAV-*TAZ* (dark hashed bars). *OMA* and *YME1L1* levels were normalized to 18S gene expression for all samples. (**I**) Bar graph showing quantified results from mitochondrial fragmentation scoring assay determining either extremely tubular, tubular, or fragmented lengths for mitochondria. (Data are presented as mean + std. err. * *p* ≤ 0.05, ** *p* ≤ 0.01, *** *p* ≤ 0.001.)

Immunofluorescence (IF) analysis of translocase of outer mitochondrial membrane 20 (TOMM20) expression in healthy and BTHS lines enabled observations of mitochondrial distributions in cells (Figure 3). In general, the mitochondria in healthy cells and in the AAV-*TAZ*-treated BTHS cells appear to be more evenly distributed throughout the cytoplasm as compared to untreated BTHS cells, where mitochondria display a more perinuclear localization (Figure 3A–F). Mitochondrial fragmentation was assessed by gene expression analyses of *YME1L1* and *OMA*—two markers of fragmentation that were significantly upregulated in both BTHS lines as compared to healthy controls (Figure 3G,H). Fragmentation gene expression levels were significantly improved in both BTHS lines following transduction by AAV-*TAZ*. Fragmentation scoring by blinded analysis enabled further comparisons of these profiles revealed increased levels of fragmented and decreased levels of tubular mitochondria in both of the BTHS lines as compared to healthy controls. These fragmented mitochondrial profiles were significantly improved following treatment with AAV-*TAZ* in both BTHS lines, as shown in Figure 3I and Figure S1.

### 2.4. Impact of AAV-*TAZ* Treatment on Mitochondrial Function

**Figure 4:**
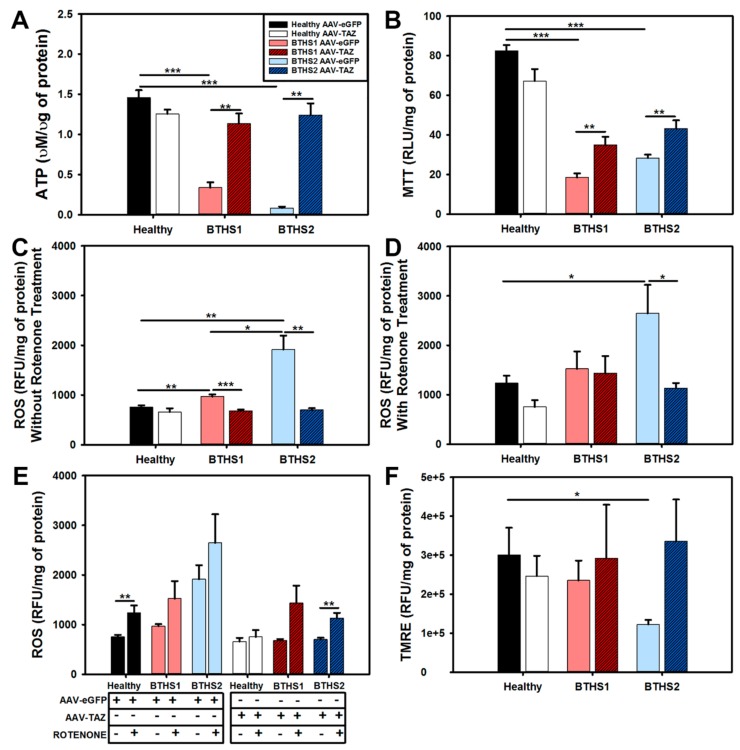
Mitochondrial function assays following AAV-*TAZ* treatment: (**A**) ATP generation, (**B**) MTT levels, (**C**) ROS levels, (**D**) ROS levels (intracellular ROS derived from superoxide O_2_^−^) following addition of rotenone. (**E**) Information from (**C**) and (**D**) combined into one graph, and (**F**) TMRE (mitochondrial membrane potential levels) relative fluorescence levels from cells following administration of control AAV-*eGFP* (light filled bars) and AAV-*TAZ* (dark hashed bars) vectors. (Data are presented as mean + std. err. * *p* ≤ 0.05, ** *p* ≤ 0.01, *** *p* ≤ 0.001.)

An intracellular ATP quantitation assay was performed to measure relative ATP generation between AAV-*TAZ* and AAV-*eGFP*-treated BTHS lines and healthy controls. The results showed decreased ATP generation in both BTHS lines that was significantly increased in AAV-*TAZ*-treated groups (Figure 4A). Next, a MTT assay was performed to further assess general metabolic activity levels in each line. The MTT assay displayed similar results with reduced activities in BTHS lines as compared to healthy controls that were significantly improved in AAV-*TAZ*-treated groups (Figure 4B). Interestingly, assessments of ROS levels (as measured by a dihydroethidium (DHE)-based assay) without rotenone showed that the significant increase in both BTHS1 and BTHS2 as compared to healthy controls was ameliorated when the BTHS lines were treated with AAV-*TAZ* (Figure 4C,E). However, when ROS levels were measured following the addition of rotenone, only the BTHS2 line showed a significant increase in ROS as compared to healthy controls, although this difference was also eliminated following AAV-*TAZ* treatment (Figure 4D,E). Evaluation of mitochondrial membrane potentials (Δψm) only revealed a significant difference between BTHS 2 and healthy cells without AAV-*TAZ* treatment (Figure 4F).

**Figure 5:**
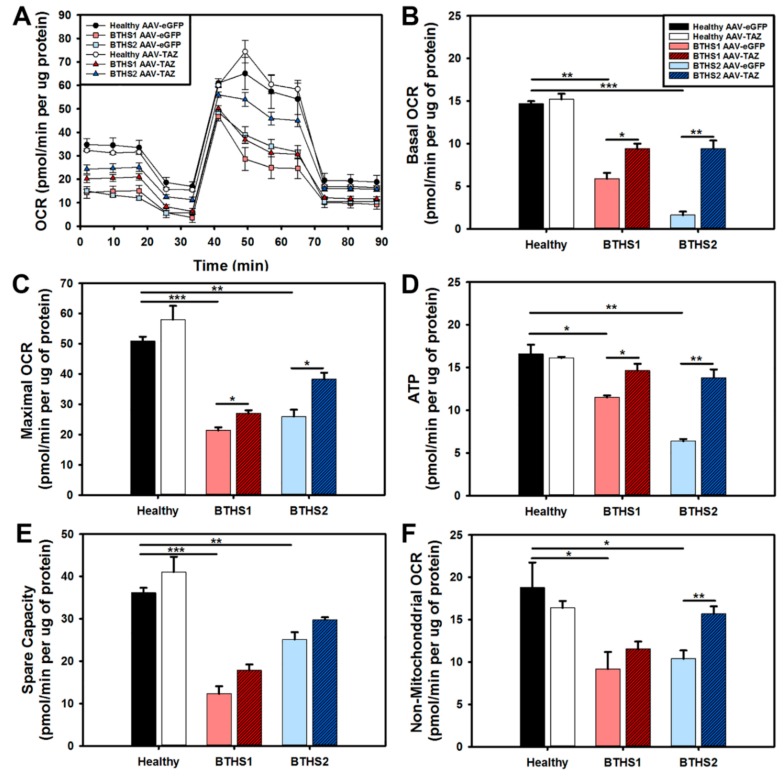
Seahorse extracellular flux mitochondrial stress test assay: (**A**) Profiles from all lines, (**B**) basal OCR, (**C**) maximal OCR, (**D**) ATP generation, (**E**) spare capacity, and (**F**) non-mitochondrial OCR from healthy controls and BTHS fibroblasts treated with either control AAV-*eGFP* (light filled bars) or AAV-*TAZ* (dark hashed bars). (Data are presented as mean + std. err. * *p* ≤ 0.05, ** *p* ≤ 0.01, *** *p* ≤ 0.001.)

The seahorse extracellular flux–mitochondrial stress test was used to compare oxygen consumption rates (OCR) between healthy and BTHS cells following treatment with either the AAV-*eGFP* control or the AAV-*TAZ* therapeutic vectors (Figure 5A). The assay showed significant improvements in basal and maximal OCR in both BTHS lines following AAV-*TAZ* treatment (Figure 5B,C). As observed in the intracellular ATP quantitation assay, a significant increase in ATP production was also observed in both BTHS lines following treatment with AAV-*TAZ* using the seahorse mitochondrial stress test (Figure 5D). Both spare capacity and non-mitochondrial OCR ceased to be significantly decreased as compared to healthy controls following treatment with the AAV-*TAZ* vector (Figure 5E,F).

## 3. Discussion

This study demonstrates that the reduced mtDNA copy numbers observed in primary BTHS patient dermal fibroblasts can be efficiently recovered following AAV-mediated *TAZ* gene replacement. As BTHS is primarily a disorder of IMM instability, these cells provide a unique mitochondrial environment in which to assess mtDNA levels. The use of two BTHS lines representing mutations of different severity in the *TAZ* locus (BTHS1—missense and BTHS2—a deletion resulting in a frameshift and early stop codon) provides useful information regarding the potentially distinct impact of various mutations on mitochondrial function as well as their respective responses to gene replacement. In addition to differences in genetic severity, these primary fibroblast cells were derived from BTHS patients who represent the phenotypic variability that exists in the BTHS population. The BTHS1 cells were isolated from a clinically mild patient who presented with decompensated cardiomyopathy during the neonatal period that responded well to digoxin for one year and has had no further cardiac disease for 40+ years. In contrast, the BTHS2 cells are derived from a patient who presented with dilated cardiomyopathy, neutropenia, 3-methylglutaconic aciduria, and skeletal muscle weakness [6,17,18].

As there is a critical need for the advancement of BTHS therapies, we have been developing an AAV-*TAZ* gene replacement strategy. AAV is optimal for gene therapies designed to treat single gene defects because the recombinant AAV (rAAV) used for gene delivery is a relatively nonpathogenic virus (with all viral genes removed) that elicits a minimal immune response. Another attribute is its long-term persistence as an episome within the nucleus of cells providing stable gene expression without risk of insertional mutagenesis [22]. We have previously demonstrated successful in vivo AAV serotype 9 delivery of *TAZ* in a mouse model of BTHS that resulted in significant improvements in mitochondrial function, heart and skeletal muscle function, whole-body activity levels, and global cardiac proteomic profiles [19,20]. For this in vitro study, we took advantage of an AAV capsid we had previously demonstrated to efficiently transduce primary canine dermal fibroblasts [23]. This AAV2 capsid’s Y–F mutations enable more efficient transduction in vitro due to the lack of capsid ubiquitination and improved intracellular trafficking to the nucleus [24].

Demonstrating the ability of our AAV-*TAZ* vector to restore mtDNA copy numbers in both BTHS patient-derived fibroblast lines further validates AAV-mediated delivery of *TAZ* as a relevant therapy for BTHS. Although many aspects involved in the response of mtDNA levels to various forms of disease or stress states remain to be defined, one protein that may be of particular importance for disorders impacting cardiac function is TMEM65. We recently discovered a significant downregulation of TMEM65 protein levels in a multiplex, global proteomics study performed on hearts from a mouse model of BTHS [20]. Other investigators have suggested that this protein (1) plays an important role in the mitochondrial inner membrane where its expression is important for mitochondrial respiration and maintenance of mtDNA copy numbers, and (2) is directly involved in the regulation of cardiac function through Cx43 function in cardiomyocytes [25,26,27]. While more research is needed to fully understand the contribution of TMEM65 levels to disease development, it is promising that in both the mouse model of BTHS and the BTHS patient fibroblast study described here, we observed increased TMEM65 levels following AAV-*TAZ* gene delivery that correspond well with various functional improvements [19,20].

Our evaluations demonstrated successful reduced expression of mitochondrial fragmentation genes (*YME1L1* and *OMA*) as well as a significant decrease in mitochondrial fragmentation. As increased levels of *YME1L1* and *OMA* are associated with upregulation of mitochondrial fission and fragmentation in response to various forms of cellular stress, successful downregulation of these genes represents another positive downstream effect stemming from improved mitochondrial structure following AAV-*TAZ* administration.

Significant improvements in ATP generation were observed in AAV-*TAZ-* treated cells as demonstrated by both an intracellular ATP quantitation assay as well as calculated from seahorse extracellular flux–mitochondrial stress tests. Finally, as a strong indication of overall improved mitochondrial health, we found that AAV-*TAZ* administration improved basal and maximal mitochondrial respiration in BTHS cells.

In sum, this study shows the ability of *TAZ* gene replacement to dramatically improve mitochondrial structure, mtDNA copy numbers, and mitochondrial function in human BTHS cells representing *TAZ* mutations of varied severity and supports further translation of AAV-mediated gene therapy for BTHS into the clinical realm.

## 4. Methods

Fibroblast Culture Maintenance: De-identified human fibroblasts were approved for use under IRB201400246. For maintenance, cells were grown at 37 °C in 5% CO_2_ in DMEM (Corning – Corning, NY, USA 10-013CV) + 20% Fetal bovine serum (FBS) and 1% Pen/Strep.

EM Processing and Analyses: Fibroblasts were seeded onto round glass coverslips and grown for two days. They were then rinsed in PBS 3× and placed in in Karnovsky’s fixative (2% paraformaldehyde/2.5% glutaraldehyde in 0.1 M sodium cacodylate buffer) and maintained at 4 °C until processing. Sample embedding, sectioning, and scanning electron microscopy (SEM) were performed by the Emory University Robert P. Apkarian Integrated Electron Microscopy Core. All measurement analyses were performed by laboratory personnel blinded to sample identities using ImageJ software (NIH). Circularity was calculated as 4πArea/Perimeter^2^. Aspect ratios were calculated as major axis/minor axis. Roundness was calculated as 4*Area/(πMajor Axis^2^). Solidity was calculated as Area/Convex Area. *n* ≥ 50 mitochondria from a total of at least 10 EM images for each line.

AAV Mediated Transduction: a multiplicity of infection (MOI) of 50,000 viral genomes (v.g.) per cell. They were incubated for seven days with medium changes every other day. Cells were then detached, centrifuged at 49 *g* for 5 min, supernatant removed and pellets stored at −80 °C.

Taqman Gene Expression Analysis: Total RNA was isolated using Quick-RNA Mini-prep kit (Zymo Research, Irvine, CA, USA) according to the manufacturer’s protocol. The gDNA was removed using DNase I (Zymo Research). Quantitative real-time (RT)-PCR was performed on an Applied Biosystems (Foster City, CA, USA) Step-One Plus Real-Time PCR System using Taqman primer/probes designed (Table 2).

Relative mtDNA copy number: gDNA was isolated using a Quick-gDNA Mini-prep kit (Zymo Research) according to the manufacturer’s protocol. Quantitative real-time (RT)-PCR was performed on an Applied Biosystems Step-One Plus Real-Time PCR System using Taqman primer/probes designed based upon previous studies (Table 3) [28,29].

Immunofluorescence: Representative fibroblasts from each line and condition were seeded onto glass coverslips and grown for 24 h. They were then rinsed three times in 1X PBS, fixed with 4% paraformaldehyde, and maintained at 4 °C until processing. They were stained with TOMM20 anti-Rabbit (HPA011562, 1:200; Sigma-Aldrich, St. Louis, MO, USA) and Phalloidin (A12379, 1:200; ThermoFisher - Life Technologies, Waltham, MA, USA) and mounted on microscope slides for imaging with medium containing DAPI. All fragmentation analyses were performed by laboratory personnel blinded to sample identities using ImageJ software (NIH). Fragmentation scores were calculated as length of ≥ 30 mitochondria in each of 10 IF images for every line. The mitochondria were scored as either fragmented (0–2 μM), tubular (2–3), or extensively tubular (3.5–5.0) (Table 4).

Determination of Mitochondrial Membrane Potential (TMRE – T669 ThermoFisher - Life Technologies, Waltham, MA, USA): Mitochondrial membrane potential was measured using tetramethylrhodamine methyl ester (TMRE). Cells were seeded at 30,000 cells per well in a 96-well black bottom plate. After 24 h TMRE dye was added to the medium at 250 nM final concentration for 30 min. The membrane potential was normalized to total protein.

Intracellular ATP Quantification (ATPlite kit – 6,016,941 Perkin Elmer, Waltham, MA, USA): Intracellular ATP production was measured by the ATPlite kit according to the manufacturer’s protocol. ATP production was normalized to total protein as measured by DC protein assay kit for each sample.

MTT Assay (M2128 ThermoFisher - Life Technologies, Waltham, MA, USA ): The methyltetrazolium bromide assay was used to determine mitochondrial dehydrogenase activity. Cells were seeded at 30,000 cells per well in a 96-well clear bottom plate. After 24 h MTT dye was added to the medium at 5 mg/mL for 2 h. The dehydrogenase activity was normalized to total protein.

Determination of Reactive Oxygen Species (D23107 ThermoFisher - Life Technologies, Waltham, MA, USA ): Dihydroethidium (DHE) was used to determine reactive oxygen species production (intracellular from superoxide) as previously described [23,30,31]. Cells were seeded at 30,000 cells per well in a 96-well black bottom plate. After 24 h the medium was aspirated and half the wells were treated with 10 µM of rotenone (a positive control for ROS production) and incubated for 1 h. The medium was aspirated and all wells were washed with 1X-PBS. DHE was added to the medium at a concentration of 5 µM and incubated for 20 min. ROS levels were normalized to total protein for all samples.

Extracellular Flux Assay: Cells were seeded at a density of 30,000/80 µL per well in XF96-well microplate (Seahorse Bioscience, Billerica, MA, USA) pre-coated with 25 µL per well of 0.1% gelatin. Cells were incubated for 24 h into standard growth medium in a humidified incubator at 37 °C with 5% CO_2_. After 24 h, the standard medium was exchanged by XF Base Medium pH 7.4 (Seahorse Bioscience) supplemented with 25 mM glucose, 2 mM L-glutamine, and 1 mM sodium pyruvate. Subsequently, were incubated for 30 min at 37 °C without CO_2_. OCR and ECAR were determined using XF Cell Mito Stress Assay (Seahorse Bioscience) following additions of: (1) ATP synthase inhibitor: 1 µM oligomycin, (2) uncoupler: 1 µM carbonyl cyanide 4-(trifluoromethoxy)phenylhydrazone (FCCP) and (3) Complex I/II inhibitors: 0.5 µM Rotenone/Antimycin A. Data were analyzed using Wave Desktop Software (Seahorse Bioscience) following the manufacturer’s instructions and normalized to protein levels.

Data Processing: Data analyses, graph creation, and statistical analyses were performed using Sigma Plot Software. There were four biological replicates for each condition and experiment and each biological sample was tested in triplicate. Descriptive statistics and paired *t*-tests were performed on all data.

## Figures and Tables

**Table 1:** BTHS mutations.

DNA Mutation:	Exon:	Amino Acid:	Mutation Type:
c.170G > T	Exon 2	p.Arg57Leu	Missense
c.140-152del13	Exon 2	p.Arg47Thr fs*32	Frameshift

**Table 2:** Primers used in quantitative RT-PCR.

Gene		Catalog	Company
18S ribosomal RNA	18S	Hs03003631-g1	Thermo Scientific
Tafazzin	TAZ	Hs00794094_m1	Thermo Scientific
YME1 like 1 ATPase	YME1L1	Hs00907919-m1	Thermo Scientific
OMA1 zinc metallopeptidase	OMA1	Hs00377028-m1	Thermo Scientific

**Table 3:** Primers used in quantitative RT-PCR.

Gene		Catalog	Company
AB mitochondrial gene 7S, encoding D-loop, which is a replication start site of the mtDNA	MT-7S	HS02596861_s1	Thermo Scientific
Hemoglobin subunit beta	HBB	HS00758889_s1	Thermo Scientific

**Table 4:** Antibodies used in immunohistochemistry (I).

Protein		Dilution (I)	Company
Translocase of Outer Mitochondrial Membrane 20 kDa protein	TOMM20	1:200	Sigma Aldrich #HPA011562
Phalloidin	Alexa Fluor 488	1:200	Life Tech # A12379

## Supplementary Materials

Supplementary materials can be found at https://www.mdpi.com/1422-0067/20/14/3416/s1.
