# Supplementary material for: Increased mtDNA Abundance and Improved Function in Human Barth Syndrome Patient Fibroblasts Following AAV-TAZ Gene Delivery
Source: Int J Mol Sci. 2019 Jul 11;20(14):3416. doi: 10.3390/ijms20143416 (PMC6678701; doi:10.3390/ijms20143416)
Supplement: Supplementary file 1 [file ijms-20-03416-s001.pdf]

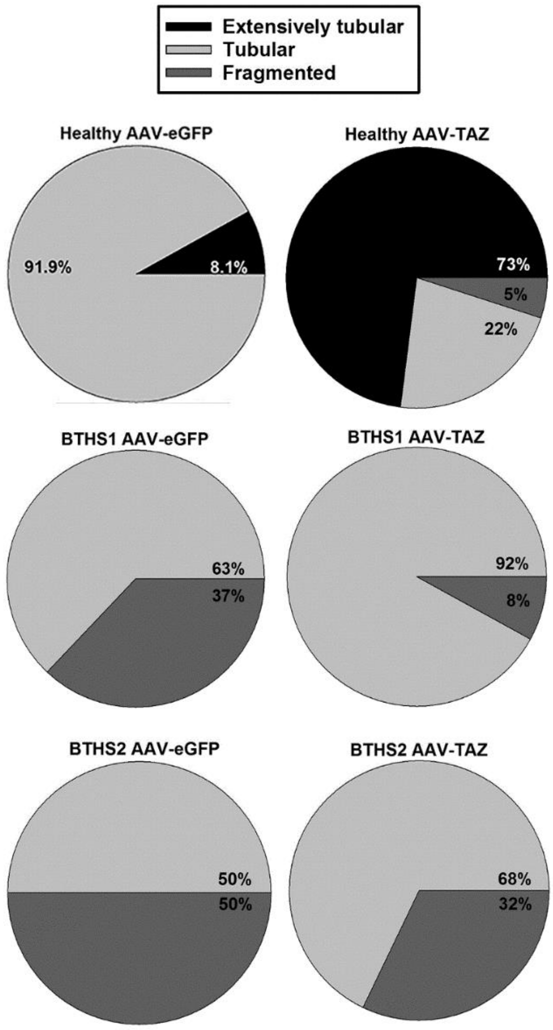

Figure S1: Pie charts displaying distribution of mitochondrial fragmentation scores in AAV-*eGFP* control and AAV-*TAZ* treated cells.
